# RISK FACTORS FOR SURGICAL WOUND INFECTION AFTER ELECTIVE LAPAROSCOPIC CHOLECYSTECTOMY

**DOI:** 10.1590/0102-672020220002e1675

**Published:** 2022-08-26

**Authors:** Gustavo de Oliveira GAMO, Gabriel Sebben REICHARDT, Camila Roginski GUETTER, Silvania Klug PIMENTEL

**Affiliations:** 1 Universidade Federal do Paraná, Departament of Surgery – Curitiba (PR), Brazil.

**Keywords:** Cholecystitis, Cholelithiasis, Cholecystectomy, Laparoscopy, Wound Infection, Colecistite, Colelitíase, Colecistectomia, Laparoscopia, Infecção dos Ferimentos

## Abstract

**BACKGROUND:**

One of the ways to avoid infection after surgical procedures is through antibiotic prophylaxis. This occurs in cholecystectomies with certain risk factors for infection. However, some guidelines suggest the use of antibiotic prophylaxis for all cholecystectomies, although current evidence does not indicate any advantage of this practice in the absence of risk factors.

**AIMS:**

This study aims to evaluate the incidence of wound infection after elective laparoscopic cholecystectomies and the use of antibiotic prophylaxis in these procedures.

**METHODS:**

This is a retrospective study of 439 patients with chronic cholecystitis and cholelithiasis, accounting for different risk factors for wound infection.

**RESULTS:**

There were seven cases of wound infection (1.59%). No antibiotic prophylaxis regimen significantly altered infection rates. There was a statistically significant correlation between wound infection and male patients (p=0.013). No other analyzed risk factor showed a statistical correlation with wound infection.

**CONCLUSIONS:**

The nonuse of antibiotic prophylaxis and other analyzed factors did not present a significant correlation for the increase in the occurrence of wound infection. Studies with a larger sample and a control group without antibiotic prophylaxis are necessary.

## RESUMO

**RACIONAL:**

Uma das formas de evitar infecção após procedimentos cirúrgicos é por meio de profilaxia com antibióticos. Isso ocorre em colecistectomias com certos fatores de risco para infecção. No entanto, algumas diretrizes sugerem o uso de antibioticoprofilaxia para todas as colecistectomias, embora evidências atuais não indiquem benefício dessa prática na ausência de fatores de risco.

**OBJETIVOS:**

Avaliar a incidência de infecção em ferida operatória após colecistectomias laparoscópicas eletivas e o uso de antibioticoprofilaxia nesses procedimentos.

**MÉTODOS:**

Estudo retrospectivo de 439 pacientes com colecistite crônica e colelitíase, contabilizados os diferentes fatores de risco para infecção de ferida operatória.

**RESULTADOS:**

Ocorreram sete casos de infecção de ferida operatória (1.59%). Nenhum esquema de antibioticoprofilaxia alterou significativamente as taxas de infecção. Foi registrada correlação estatística significativa entre infecção de ferida operatória e pacientes do sexo masculino (p=0.013). Nenhum outro fator de risco analisado demonstrou correlação estatística com infecção de ferida operatória.

**CONCLUSÕES:**

O não emprego de antibioticoprofilaxia e outros fatores analisados não apresentaram correlação significativa para aumento na frequência de infecção na ferida operatória. Estudos com maior amostra e grupo controle sem antibioticoprofilaxia são necessários.

## INTRODUCTION

Wound infection (WI) is one of the most common postoperative complications and its incidence depends on multiple factors, from the surgical and postoperative environment to the type of procedure and patient profile^4^. Cholecystectomy is one of the most performed procedures currently, and the laparoscopic method has been replacing the open procedure as it is less invasive, with a consequent lower risk of infection, shorter hospital stay, and faster recovery^32^. In our hospital service, cholecystectomy is considered a potentially contaminated procedure, with an infection risk of less than 5%^9^. According to the hospital’s Antibiotic Prophylaxis Protocol, the prophylactic use of antibiotics is recommended for open cholecystectomy and high-risk laparoscopic cholecystectomy (LC) (high-risk factors being bile spillage, acute cholecystitis or pancreatitis, jaundice, pregnancy, use of intraoperative cholangiography, conversion to open surgery, immune suppression, and prosthesis implantation)^9^. However, as certain risk factors cannot be predicted, it is customary among surgeons to perform antibiotic prophylaxis (ABP) for any cholecystectomy, even for those considered to be of low risk^1,4^.

In both literature and international guidelines, it is reported that the unnecessary use of antibiotics presents a risk of microbial resistance and generates unnecessary expenses, and their use in clean and potentially contaminated procedures does not reduce infection rates^1,16^. According to Brazil’s Price Database for Health, the average price per cefazolin (1 g) dose, the most commonly used antibiotic in ABP, is around US$ 1.28^18^. Considering the annual volume of performed procedures and the economic burden that antimicrobial therapy can represent (up to 64% of hospital pharmacological costs), the rational use of this resource is certainly beneficial^25^.

Furthermore, the influence of some risk factors on the incidence of WI is controversial, such as bile spillage during the procedure, in cases without acute inflammation or empyema^1,2,5,8,10,21,26–31^. The evaluation of these factors would allow the elimination of unnecessary indications of ABP, reducing hospital costs, and other consequences of the use of antibiotics.

This study aims to analyze the influence of different risk factors and ABP usage in the occurrence of WI after elective laparoscopic cholecystectomies.

## METHODS

A retrospective, observational study was carried out with patients diagnosed with cholelithiasis in regular follow-up at the hospital, from September 25, 2018, to October 28, 2019. The study protocol was approved by Hospital do Trabalhador’s (Worker’s Hospital) Ethics Committee under Presentation Certificate for Ethical Appreciation (CAEE) 17016619.1.0000.5225.

Clinical and laboratory data were taken from the patients’ medical records, anesthesiology documents, and anatomopathological reports. Surgical occurrences were taken from the surgery reports. Patients aged between 18 and 70 years, diagnosed with cholelithiasis and chronic cholecystitis, who underwent elective LC, and with a minimum of 10-day follow-up at the Hospital’s General Surgery outpatient clinic were included in the study.

Through the evaluation of the anesthetic records, risk factors for infection were identified in patients. The analyzed factors were as follows: sex, age, body mass index (BMI), smoking, diabetes, immunosuppression, surgical procedure within 6 months prior to cholecystectomy, infection within 30 days prior to surgery, a history of jaundice, a history of acute pancreatitis, and a risk score according to the American Society of Anesthesiologists (ASA). Through surgical and medical records, length of surgery, length of hospital stay, use of cholangiography, bile duct injury, use of ABP, and gallbladder ruptures were all analyzed. Through follow-up registered in medical records, cases of WI and complications that could require reoperation were analyzed. Risk factors were deemed, as per literature, a BMI equal to or greater than 25 kg/m^2^, an ASA score equal to or greater than 3, and surgery length greater than 2 h^1^.

Cases in which anatomopathological analysis indicated acute cholecystitis or gallbladder empyema, cases of incomplete medical records, loss of follow-up, or other procedures performed simultaneously to cholecystectomy, unrelated to bile ducts, were excluded.

Statistical analysis was performed through the use of the Stata 14.2 software. Primarily, the distribution pattern for continuous variables was evaluated using the Shapiro-Wilk normality test. For descriptive analysis, measurements of median trends and dispersion were expressed in mean and standard deviation (mean+SD). Continuous variables determined as non-normal were expressed in median and minimum–maximum values. Categorical variables were expressed as absolute and relative frequencies. For inferred statistical analysis, the Wilcoxon tests were used for continuous dependent variables and chi-squared tests for binary or categorical dependent variables. A significance level of 5% was taken into consideration for this test.

## RESULTS

Data were collected from 541 patients. After application of the criteria, 102 patients were excluded, leaving a total of 439 cases for the study, of which 79.95% were female. The data needed to calculate BMI were absent in 115 cases; therefore, the BMI data refer to only 324 patients whose average was 29.32 (±5.20).

The risk factors found were: smoking in 63 cases (14.35%), diabetes in 36 cases (8.20%), jaundice in 2 cases (0.46%), immunosuppression in 4 cases (0.91%), previous surgeries in 10 cases (2.28%), previous infection in 4 cases (0.91%), acute pancreatitis in 2 cases (0.46%), ASA score ≥3 in 16 cases (3.76%), use of intraoperative cholangiography in 6 cases (1.37%), bile duct injury in 1 case (0.23%), and gallbladder rupture in 12 cases (2.73%). The descriptive analysis and risk factors of these cases are summarized in Table 1.

**Table 1 -:** Descriptive analysis and analyzed risk factors.

Variables	Overall
Age (median, min-max) (years)	46.2 (36.2–56.8)
Male (%)	88 (20.05)
Female (%)	351 (79.95)
BMI (median, min-max) kg/m^2^	29.14 (25.71–32.38)
Smoking (%)	63 (14.35)
Diabetes (%)	36 (8.20)
Jaundice (%)	2 (0.46)
Previous surgeries (%)	10 (2.28)
Previous infection (%)	4 (0.91)
Immunosuppression (%)	4 (0.91)
Pancreatitis (%)	2 (0.46)
ASA ≥3 (%)	16 (3.76)
Length of hospital stay (median, min-max) (days)	1 (1–1)
Length of surgery (median, min-max) (min)	100 (85–115)
Cholangiography (%)	6 (1.37)
Gallbladder rupture (%)	12 (2.73)
Bile duct injury (%)	1 (0.23)
WI (%)	7 (1.59)

BMI: body mass index; ASA: American Society of Anesthesiology classification; WI: surgical wound infection.

After applying the Shapiro-Wilk test, all continuous variables analyzed (age, BMI, length of hospital stay, and length of surgery) were defined as noncontinuous and were therefore shown as median and minimum–maximum values. The median age was defined as 46.2 years (36.2–56.8), BMI was defined as 29.14 kg/m^2^ (25.71–32.38), length of surgery as 100 min (85–115), and hospital stay as 1 day (1–1). The use of ABP was also investigated, with information gathered from 418 patients. Within this group, 408 patients received ABP (97.61%). Cefazolin was used on 403 patients (96.41%), cefalexin on 3 patients (0.72%), ceftriaxone on 1 patient (0.24%), and ceftriaxone combined with metronidazole on 1 patient (0.24%). An important factor observed in this group is that 101 patients (24.16%) received ABP, regardless of presenting no risk factors for WI (excluding sex as a risk factor). The prophylactic antibiotic therapy used is described in Table 2.

**Table 2 -:** Prophylactic antibiotic therapy used in the analyzed sample.

ABP	Overall (%)
Total	418 (100)
None	10 (2.39)
Cefazoline	403 (96.41)
Cefalexin	3 (0.72)
Ceftriaxone	1 (0.24)
Ceftriaxone + metronidazole	1 (0.24)
ABP with no indication	101 (24.16)

ABP: antibiotic prophylaxis.

Cases with an outcome of WI, which required antibiotic therapy and/or reoperation, as well as other noninfectious outcomes which required reoperation, were analyzed. There were seven cases of WI. Four cases required reoperation, of which there was one case of infection, one case of biloma, one case of hematoma, and one case of biloma associated with hematoma. In statistical analysis, it was observed that the incidence of WI was significantly higher for male patients (p=0.013). Another factor identified as correlated with WI was bile duct injury. There was only a single case of injury identified, however it was followed by a case of WI (p=0.000). Among other risk factors analyzed, the correlation with WI was not statistically significant. Furthermore, there was no statistically significant difference between different ABP regimens in the incidence of WI. Of the 10 cases that did not receive ABP, none developed WI. The statistical analysis of relative risk factors is summarized in Table 3.

**Table 3 -:** Statistical analysis of risk factors for wound infection.

Variables	Total	With WI	Without WI	p-value
Age	439	–	–	0.3388
BMI	324	–	–	0.1787
Male	88	4	84	0.013
Female	351	3	348	–
Smoking	63	0	63	0.275
Diabetes	36	0	36	0.425
Jaundice	2	0	2	0.857
Immunosuppression	4	0	4	0.798
Pancreatitis	2	0	2	0.857
Previous surgery	10	0	10	0.684
Previous infection	4	0	4	0.798
Length of surgery	439	–	–	0.2210
Hospital stay	439	–	–	0.5505
ASA ≥3	16	0	16	0.388
Cholangiography	6	0	6	0.754
Gallbladder rupture	12	0	12	0.655
Lesion	1	1	0	0.000
With ABP	408	7	401	0.992
Without ABP	10	0	10	–

BMI: body mass index; ASA: American Society of Anesthesiology Score; WI: wound infection; ABP: antibiotic prophylaxis.

## DISCUSSION

LC is considered a safe procedure, comparable to clean procedures, particularly in elective cases and in patients with no risk factors^6^. Studies indicate that cholecystectomy can even be performed simultaneously with other procedures without increasing the risk of infection^3^. The incidence of infection is around 0.71–8.7%^2,5,6,8,10–12,20–22,26–32^. There are a growing number of results that do not demonstrate a significant correlation between ABP in low-risk procedures and a reduction in infection rates^2,5,7,8,19,20,24,26–28,30,33^. However, there are also studies that point to a protective effect of antibiotics, leading to uncertainty^4,14,17,23,31^.

In this study, the incidence of WI was 1.59%, which is consistent with known literature. The risk factors evaluated were chosen based on international guidelines, in addition to studies that found a significant correlation to WI^1,4–6,8,10,11,13,21,22,26,28–32^. The predominant epidemiological profile in this study was consistent with the literature, with a prevalence given to female patients aged between 30 and 50 years. A recognized infection risk, a high BMI, was prevalent in this study group (median 29.14 kg/m^2^), which can be expected as this is also a known risk factor for the development of gallstones^13^.

Among the risk factors analyzed, no significant statistical correlation was found between WI and BMI, surgery length, hospital stay, or age. This lack of correlation may be due to un-normal presentation of these factors, as there is not enough variation within such factors to show different outcomes, seeing as the relevance of these data has already been proved^1,4,6,8,13,22,30,32^. No correlation was found either for smoking, diabetes, pancreatitis, immunosuppression, prior infection or surgery, jaundice, or an ≥3 ASA score. Some of these factors are recognized in the literature as risk factors for WI, but they were not found in sufficient numbers to warrant an adequate statistical analysis^1,4,6,21,22,30,32^.

The incidence of gallbladder perforation was 2.73%. This value is in line with the lowest rates reported in the literature, which shows a large variation, i.e., between 1.5 and 35.1%^2,5,8,10–12,26–28,30,31^. It is possible that there is underreporting, as it is a common occurrence with this procedure, and may not be included in the medical records by the surgeon^30^. There was no statistically significant correlation between bile spillage and WI in cases of cholelithiasis with no acute cholecystitis, which is in accordance with other studies^2,8,10,27,30^. This finding is important when considering the recommendation for ABP, as the rupture of the gallbladder is a factor that cannot be predicted before the procedure, which could justify ABP usage in all cholecystectomies. A characteristic of some studies that did demonstrate a statistical correlation between perforation and infection is the inclusion of cases with acute inflammation and complicated cases, with a conversion to open surgery^21,29^. These same studies, in turn, showed above-average infection rates. This indicates that the risk factor may not necessarily be the bile itself, but its infection, so that a more inflamed and, therefore, more fragmentable and rupture-prone gallbladder is just an indicator of an already complicated case^11^. As for the asymptomatic colonization of the gallbladder, there are still conflicting results regarding its role in infectious risks^10,26,27,29^. It is worth pointing out that there was a case of an injured bile duct that presented a WI, which can be explained by the more aggressive intervention that may have caused the injury; however, a single isolated case cannot define a statistical correlation.

There was no significant correlation between WI and the use of ABP, as already demonstrated in the bibliography. The use of ABP has already been evaluated in multiple meta-analyses, which have not demonstrated any benefit with such practice^7,19,24,33^. Even in studies in which gallbladder rupture significantly increased the incidence of WI, prophylaxis had no protective effect^5,30^. However, the unnecessary use of antibiotics is commonplace. A study showed that 94.5% of professionals used ABP in elective LCs^15^. In our study, about a quarter of the evaluated patients received ABP, despite having no risk factors that justified this approach. Infection by *C. difficile* can represent up to 10% of surgical infections, and the use of ABP can increase the risk of this type of infection^6^. As it is an infection that is more serious and more resistant to antibiotics, the rational use of these drugs should be emphasized.

A significant risk factor related to WI was sex. This is shown in the literature, which indicates that male patients have a higher probability of having complications in surgery and getting infected^5,11,29,32^. Possible explanations for this correlation involve a greater inflammatory pattern of cholecystitis in males, variations in male anatomy that make the surgical procedure difficult, and a predisposition of male patients to seek health services less frequently than females, therefore receiving medical care at a much more advanced clinical stage^11^.

This study has limitations. The low number of cases of WI, which in itself is a rare event, makes statistical analysis difficult and hinders the study of isolated variables. Furthermore, the larger number of patients with ABP, compared to the group without ABP, precludes the presence of an effective control group to accurately assess the effectiveness of ABP. Another factor, specifically regarding gallbladder perforation, is that its incidence may be reduced by underreporting, as it depends wholly on the surgeon’s inclusion of this event in the surgical report.

## CONCLUSIONS

Patients who undergo elective LC with few risk factors do not benefit from the use of ABP. Antibiotics should be reserved for complicated and emergency cases with a high risk of infection. A larger study with a control group to assess the effectiveness of ABP is needed to further support these recommendations.
